# Supplementary material for: Nutrition status and morbidity of Ethiopian children after recovery from severe acute malnutrition: Prospective matched cohort study
Source: PLoS One. 2022 Mar 10;17(3):e0264719. doi: 10.1371/journal.pone.0264719 (PMC8912152; doi:10.1371/journal.pone.0264719)
Supplement: S1 Fig — (DOCX) [file pone.0264719.s001.docx]

S1_ Fig Average height-for-age Z-score and height-for-age difference evolution of the different study groups over the follow period

S2_ Fig Average height-for-age Z-score and height-for-age difference evolution of children of the different study groups who had stunting reversal by the end of the follow period
