# Supplementary material for: Nutrition status and morbidity of Ethiopian children after recovery from severe acute malnutrition: Prospective matched cohort study
Source: PLoS One. 2022 Mar 10;17(3):e0264719. doi: 10.1371/journal.pone.0264719 (PMC8912152; doi:10.1371/journal.pone.0264719)
Supplement: S1 Table — (DOCX) [file pone.0264719.s003.docx]

Supplementary table 1. Selected household characteristics of the participants by study group

|  | Post-SAM  (n=202) | Non-wasted control (n=203) | P-value |
| --- | --- | --- | --- |
|  | n (%); median (IQR)^a^ | n (%); median (IQR) |  |
| At least one parent alive | 176 (89.8) | 194 (99.5) | <0.001 |
| Caregiver, mother | 176 (87.6) | 199 (98.5) | 0.001 |
| Age of the caregiver in years | 28(25, 34) | 25(22, 30) | 0.002 |
| Care giver ever attended school | 29 (14.4) | 39 (19.4) | 0.176 |
| Male headed household | 189 (94.0) | 194 (96.5) | 0.240 |
| Household head ever attended school | 54 (26.7) | 62 (31.2) | 0.329 |
| Household size | 6(5,8) | 5.5(4,7) | 0.020 |
| Number of children <5 years | 2(1, 2) | 2(1,2) | 0.001 |
| Water, sanitation and hygiene index |  |  | 0.064 |
| Better | 102 (50.2) | 120 (59.4) |  |
| Poor | 101 (49.8) | 82 (40.6) |  |
| Infant and child feeding index |  |  | 0.179 |
| Lowest | 8 (3.9) | 7 (3.5) |  |
| Medium | 94 (46.3) | 76 (37.6) |  |
| Highest | 101 (49.8) | 119 (58.9) |  |
| Household food security |  |  | <0.001 |
| Food secure | 117 (57.6) | 155 (76.7) |  |
| Mildly food insecure | 6 (3.0) | 7 (3.5) |  |
| Moderately food insecure | 18 (8.8) | 18 (8.9) |  |
| Severely food insecure | 62 (30.5) | 22 (10.9) |  |
| Wealth quartiles |  |  |  |
| 1^st^ | 50 (26.3) | 23.6 | 0.623 |
| 2^nd^ | 22.6 | 27.5 |  |
| 3^rd^ | 24.2 | 25.8 |  |
| 4^th^ | 26.8 | 23.1 |  |
| Maternal mid-upper arm circumference (cm) | 22.1(21.1, 23.4) | 22.8(21.6, 24.0) | 0.008 |
| ^a^IQR, Inter quartile range | | | |
