# Supplementary material for: Nutrition status and morbidity of Ethiopian children after recovery from severe acute malnutrition: Prospective matched cohort study
Source: PLoS One. 2022 Mar 10;17(3):e0264719. doi: 10.1371/journal.pone.0264719 (PMC8912152; doi:10.1371/journal.pone.0264719)
Supplement: Attachment — Submitted filename: Post-SAM_subm (edited).docx [file pone.0264719.s005.docx]

Nutrition status and morbidity of Ethiopian children after recovery from severe acute malnutrition: Prospective matched cohort study

Tsinuel Girma^1^*, James T James ^2, #a^, Abdissa Abdissa^3,#a^, Hanqi Luo^4, #a^, Yesufe Getu^5^, Yilak Fantaye^6^, Kate Sadler^7^, Paluku Bahwer ^8^.

^1^Department of Pediatrics and Child Health, Jimma University, Jimma, Ethiopia

^2^Valid International, 35 Leopold Street, Oxford OX4 1TW, United Kingdom.

ORCID: 0000-0001-5448-8193

^3^Department of Laboratory Sciences, Jimma University, Jimma, Ethiopia

4Valid International, 35 Leopold Street, Oxford OX4 1TW, United Kingdom. Orcid: 0000-0001-6253-5818

^5^Department of Physiology, Jimma University, Jimma, Ethiopia

^6^Alameda County Public Health Department, 1000 Broadway, Oakland, CA 94607

^7^Valid International, 35 Leopold Street, Oxford OX4 1TW, United Kingdom.

^8^ Valid International, 35 Leopold Street, Oxford OX4 1TW, United Kingdom.

^#a^ Armauer Hansen Research Institute, Addis Ababa, Ethiopia

^#a^ Emergency Nutrition Network, 69 High St, Marlborough House, Kidlington, OX5 2DN, United Kingdom.

^#a^ Department of Global Health, Rollins School of Public Health, Emory University

1518 Clifton Rd, Atlanta, GA 30322,USA

^#a^ Center for Epidemiology, Biostatistics, and Clinical Research, School of Public Health, Free University of Brussels, Brussels, Belgium.

*Corresponding author

Email: [tsinuel@yahoo.com](mailto:tsinuel@yahoo.com) (TG)

Competing interest: The authors have declared that no competing interests exist.

# Abstract

After recovery, children with severe acute malnutrition (SAM) remain vulnerable to sub-optimal growth and malnutrition relapse. Although there is an increased interest in understanding these problems, data are scarce, and contextual factors can cause variability. We prospectively followed a cohort of Ethiopian children (215 post-SAM cases and 215 non-wasted controls), monthly for one year. The post-SAM cases were: age 6-59 months at admission into the community management of acute malnutrition (CMAM) program and being successfully discharged from CMAM (MUAC>11.0cm, weight gain of 20%, absence of oedema and clinically stable for two consecutive weeks). The controls were apparently healthy children from same village who had no history of an episode of AM and were matched 1:1 to a post-SAM child by age and sex. The primary outcomes were: cumulative incidence of AM; growth trajectory; cumulative incidence of reported common morbidities, and cumulative proportion and incidence of deaths. The burden of common morbidities was higher (p<0.001) among post-SAM than controls; post-SAM children had more frequent illness episodes. The prevalence of SAM was consistently higher among post-SAM than the control group having a 15 times higher risk of developing SAM. The divergence in weight and growth trajectory remained the same during the study period. Our results advocate for the design of post-discharge interventions that aim to prevent the reoccurrence of acute malnutrition, reduce morbidity and promote catch-up growth. Research is needed to define the appropriate package of post-discharge interventions.

# Introduction

The community management of acute malnutrition (CMAM) approach has increased the access and coverage of treatment for severe acute malnutrition (SAM) and moderate acute malnutrition (MAM) in most low- and middle-income countries (LMIC), including Ethiopia (1-4). Remarkable results have been achieved with reports of recovery in children as high as 80% (5-8). After nutritional therapy, however, recovered children remain at risk of relapse to MAM or SAM as they often return to the same living condition that does not support optimal growth (réf???). Although there is an increased interest in understanding relapse, data are scarce, and the published reports are difficult to compare as there is no standardized definition of relapse and universally agreed indicators(9).

Anthropometric criteria and the absence of nutritional oedema are currently used as proxies of physical and physiological recovery from SAM. The treatment ends when children have corrected their body mass deficit as measured by the weight-for-height index or mid-Upper Arm Circumference (MUAC) of ≥-2 Z-score or ≥12.5 cm, respectively and/or they have no clinically detectable nutritional oedema (10). Data are lacking to confirm if these children retain the weight and MUAC velocities that allowed the rapid correction of wasting and a return to their normal growth track. Information is also needed on the effect of the correction of wasting on the subsequent linear growth.

In children who have re-gained their body mass deficit, the complex immune dysfunctions associated with SAM are expected to improve(11, 12). However, measuring the immune markers is methodologically challenging, and interpretation of the data generally is not straightforward(13). Thus, the prevalence and incidence of common morbidities are often used as proxy measures of immune recovery.

The limited available evidence suggests that relapses are common and linear growth catch-up is insufficient (9, 14-17). However, the picture may vary from one country to another, and studies are needed to confirm this. Therefore, this study aimed to assess children's nutritional status and morbidity after recovery from SAM.

# Methods

## Study design, population and data collection

This prospective matched cohort study was conducted from the September 2013 to September 2015 in the rural population of Jimma Zone, Oromiya region in Southwest Ethiopia. Of the 18 districts (woredas) in the Jimma zone, three woredas (Dedo, Omonada, and Seka) were selected purposefully due to their high caseload of SAM and accessibility. We based our sample size estimation on the following assumptions: We projected an 8.5 difference between post-SAM and controls in incidence of acute malnutrition (AM), anticipating that over the one year follow up 13.1% of post-SAM children will have experienced an episode of SAM (95% confidence interval of ±2.5 %: CI limit 7.5 % to 12.5 %).
This was based on the prevalence of SAM in Oromiya Region reported in the 2011 Ethiopian Demographic and Health Survey report (18). With an assumed design effect of 1.5 and a loss to follow up of 20%, the required sample size was 474 (237 post-SAM and 237 controls) for a power of 80% and an alpha level of 5%. For the post-SAM cases, the inclusion criteria were: age 6-59 months at admission into the CMAM program and being successfully discharged from CMAM as per the national guideline criteria at the time (MUAC>11.0cm, weight gain of 20%, absence of oedema and clinically stable for two consecutive weeks). Controls were eligible if they were apparently healthy with no history of an episode of AM and were matched 1:1 to a post-SAM child by age and sex. Mother/caregiver’s consent for their children to participate and residence in the study catchment area for at least one year after enrolment were additional criteria for both groups. We excluded children with physical disability and any congenital disease that affected growth or prevented accurate anthropometric measurement (both groups), discharged directly from the inpatient nutrition rehabilitation unit for post-SAM, and presence of acute malnutrition (MUAC<12.0 cm or bilateral pitting pedal oedema) for the control group.

Both post-SAM and control groups were followed up concurrently at their homes monthly by trained data collectors over one year or until the participants died, decided to withdraw or move out of the area. Weight, length/height, and MUAC were measured according to WHO (to be defined) standards and in duplicate(19). Length/height was measured to the nearest 0.1 cm using the UNICEF (to be defined) recommended portable wooden length/height board with an upright wooden base and movable headpiece. Children younger than 24 months were measured in supine position, and older children were measured while standing. Weight was measured to the nearest 0.1 kg using mother and child battery-powered SECA weighing scales (SECA 874, Hamburg, Germany). Younger children were weighed undressed while being held by their mothers and older children stepped on the scale. MUAC was measured at the midpoint of the left arm, using a non-stretch insertion tape to the nearest 0.1cm.

Morbidity data were collected using the two-week recall technique and are based on the caregiver's report of fever, diarrhoea, persistent cough and fast breathing during the two weeks prior to the home visit. Trained research nurses conducted voluntary HIV (to be defined) testing and counselling for all children of both groups.

Relapse of malnutrition was defined as follows: AM, MUAC <12.0 cm or pitting pedal oedema); moderate acute malnutrition (MAM), MUAC 11.0-12.0 cm and SAM, MUAC <11.0 cm or pitting bipedal oedema. The cut-offs values were based on the Ethiopian protocol that was used at the time of the study (20). We did not restrict the definition of relapse to a fixed time interval after recovery from SAM, but included all relapse cases within our study period.

## Study outcomes

The primary outcomes of interest were: cumulative incidence of AM, MAM and SAM; growth trajectory (weight length/height, MUAC and related indices changes and trends); cumulative incidence of reported common morbidities, and cumulative proportion and incidence of deaths.

## Statistical analysis

Indices for weight-for-height (WHZ), length/height-for-age (HAZ), weight-for-age (WAZ), length/height-for-age difference (HAD) and weight-for-age difference (WAD) were calculated according to WHO (2006) growth standards(21). WHZ, HAZ and WAZ were calculated using the zscore 06 command in Stata(21, 22). HAD and WAD were calculated using the approach proposed by Leroy et al. (23). HAD and WAD are the actual deficit when the child is compared to the median child of the same age and sex. We obtained a z-score by dividing this value by the corresponding standard deviation (SD). Because the SD is not a constant, this division introduces a mathematical artifact. It has been clearly demonstrated that HAD better captures the magnitude of the absolute deficit in children than HAZ and that HAZ may give a false impression of growth catch up(23). WHZ, HAZ and WAZ at enrolment were categorized into <-2 z-scores, ≥-2 to define absence or presence of wasting, stunting, and underweight, respectively. Both HAZ and HAD were used to characterize linear growth better. Based on the literature, accelerated linear growth (ALG) was diagnosed when HAZ increased by ≥0.67 between two assessments (23-26). The covariant for outcome of wasting and morbidity was assessed by fitting mixed-effects models to include independent variables as covariates. A linear mixed-effects model was applied for the continuous outcome measures (rates of WHZ, WAZ, HAZ, BAZ and MUAC changes) and time to relapse of MAM and SAM. Time to malnutrition relapse was analyzed using the Cox proportional hazard models and Kaplan Meir survival analysis. All P values are two tailed, and statistical significance was set at p-value less than 0.05 with 95% CI.

## *Ethical considerations*

The ethical review board of Jimma University approved the study (reference RPGC/130/2013). Written informed consent was obtained from the caregiver of all participants. During the follow-up, children who developed SAM at any time were referred to the nearest health facility for appropriate dietary and medical management.

# Results

The initial sample size of 474 was not reached within the planned time of recruitment. However, the loss to follow-up was two-fold lower than anticipated, making the actual sample size of 430 (215 post-SAM cases and 215 non-wasted controls) sufficient to maintain the study power. Of the 430 enrolled children, follow-up data for analysis were available for 94.4% (203/215) post-SAM and 93.9% (202/215) controls (Fig 1).

As shown in Table 1, no difference was observed between post-SAM and control groups for matching criteria at baseline except that the post-SAM children were significantly lighter and shorter (p<0.001). Household characteristics were mostly comparable between the two group (supplementary table 1)**.** All participants had an HIV-negative test result.

In the post-SAM, the prevalence of MAM decreased from 7.5% to 5% by the end of the six-month follow-up (Fig 2). Meanwhile, the point prevalence of SAM (monthly) was consistently higher among post-SAM than the control group, with peaks at the third (3.6%) and seventh month (3.8%). In contrast, the control group had only two SAM cases at the eleventh month. Over the year, the post-SAM group had 15 times the risk of developing SAM than controls (table 2). There were six deaths, all from post-SAM; six of them had AM (two with SAM). All deaths except one were disease-related, which was due to accident (data not shown).

As table 3 indicates, the post-SAM group were 1.4 times likely to have any of the common morbidities (cough, diarrhoea, fever or difficulty breathing) compared with the controls during the one year follow-up period (p<0.001). Whereas, the risk for the individual morbidities was at least 1.7 times in the post-SAM compared to controls (p<0.001).

The burden of common morbidities was higher (p<0.001) among post-SAM than controls (Fig 3); post-SAM children had more frequent illness episodes. On the other hand, the proportion of children who never reported the symptoms were lower for post-SAM children than non-wasted controls.

Table 4 shows anthropometric parameters at baseline and their changes over 12 months. Although the post-SAM group were discharged from CMAM as recovered, they were still significantly lighter, shorter, more wasted and more stunted than controls, both at baseline and 12 months later. The post-SAM group were less wasted at 12 months than at baseline but compared to controls they did not significantly reduce their deficit in weight, or MUAC by the 12^th^ months follow up, except when WAZ is considered. At 12 months, both the cases and controls were more stunted than at baseline, with more pronounced drop in linear growth in the controls but this translated in a significant reduction of the deficit in length/height only when the HAZ was considered and not when absolute length/height or HAD are used for comparison. The indices WAD and HAD showed that the widening of the deficit in weight and length/height was larger than depicted by the indices WAZ and HAZ and showed weight deficit widening even among post-SAM for whom WHZ and WAZ showed some catch up.

Figure 4 presents the overall trend of the anthropometric parameters during the 12 months of follow-up. The differences in weight, height and MUAC between post-SAM and controls did not reduce or widen during the follow up though WAZ and WHZ differences were slightly decreasing over time. Both HAZ and HAD curves showed that the post-SAM group did not experience catch-up linear growth compared to the control group and the age and sex corresponding WHO 2006 medians (supplementary Fig 1). However, a quarter of post-SAM children [40/156=25.6 (18.7; 32.6) %] had a HAZ increase of ≥0.67 by the 12^th^ month follow-up, meeting the definition of ALG. Among post-SAM who were stunted at baseline, stunting reversal was observed in 14.4 % [18/125=14.4 (8.1; 20.6) %]. The figure was 22.9 % [11/148=22.9 (10.6; 35.2) %] among the controls who were stunted at baseline. Both HAZ and HAD had an upward trend for those children who reversed stunting (supplementary Fig 2)

# Discussion

This study is the first to concurrently follow up post-SAM children and matched controls for an extended period after recovery from the index episode of SAM. It has shown that the post-SAM group remained at higher risk of AM and common childhood disease occurrence than controls for a period as long as 12 months after recovery and that catch-up growth does not occur, though a complete stunting reversal is observed in a small proportion of children stunted at enrolment. These results can significantly contribute to the design of post-SAM interventions.

The recurrence of AM after recovery has been the focus of several publications recently(9, 16, 27). Thus, there is now sufficient evidence to suggest that post-SAM children need special attention, including sustained follow-up and correction of persisting deficits even when they have recovered based on anthropometry(13, 28, 29). This study has shown that this special attention is required throughout the 12 months after recovery as vulnerability to AM and infection persisted throughout the 12 months follow-up period. Other studies have shown a shorter period of vulnerability, indicating that in some contexts, a short 3-month period of post-discharge care may be sufficient(9, 16). However, in addition to the timing of AM recurrence, other parameters should be considered for the decision on the optimal period of post-discharge follow-up, including immune system reconstitution, correction of anaemia, and other micronutrient deficiencies. Whilst some studies have shown that by the time children reach anthropometric discharge criteria, most nutritional and immune system (measured by thymus size) markers have returned to normal, other studies have shown that deficiencies remain(12, 13, 28-31).

In addition to defining the duration of post-discharge follow-up, there is also a need to define the package of interventions to support sustained recovery and restore normalcy of all physiological functions. Currently, evidence is lacking as recently tested post-discharge interventions, including infection control, food supplementation, micronutrient supplementation, and a combination of these approaches, have yielded inconclusive results(32, 33). However, assuming current research efforts are sustained, including testing new interventions such as those to address maternal mental disorders or environmental enteric dysfunction, there are chances that a package of post-treatment support to prevent the recurrence of acute malnutrition will be available shortly. (34-36).

If post-recovery follow-up were to be introduced in the management of SAM, potential options include either integrating follow-up into a routine Growth Monitoring and Promotion (GMP) programme or adding follow-up phase to the CMAM protocol. Our results advocate for the former, as such integration will allow a longer surveillance of these post-SAM children. Indeed, it may be challenging to manage the extra workload that an additional CMAM follow-up phase would require in many high-burden countries.

Correction of body mass deficit is among the primary objectives of SAM treatment (10, 37, 38). Based on the comparison of post-SAM and controls we can conclude that this objective was only partially reached. Whilst most post-SAM children met the treatment discharge criteria recommended at the time and grew at the same speed as the controls, the body mass of these children remained smaller than that of matched controls. Moreover, no catch-up trend was observed during the subsequent 12 months. However, this conclusion is valid only if Post-SAM had a growth trajectory similar to that of controls prior to developing the episode of SAM for which they were admitted. There is a body of evidence that prenatal and early postnatal factors may lead to programmed growth restriction during childhood and even up to adulthood (39, 40). Our results showing similarity of growth velocity between the Post-SAM and the controls during the follow-up period leads to the conclusion that they were growing well and suggests that the lack of catch up is unlikely to be due to factors operating during that period. The observed weight and length/height increments and the overall growth profiles allow us to formulate two complementary hypotheses. First, SAM disproportionally affects children by restricting growth along trajectories that follow the lower percentiles of the WHO 2006 growth curves. Second, by the time of graduation from the treatment programme, post-SAM had already corrected their growth deficits and subsequently grew as per their prenatally, and early infancy programmed growth trajectories. The first hypothesis is difficult to test as data on birth weight or growth prior to developing SAM were not available. But it is worth mentioning that in both Post-SAM and non-wasted control groups, children who experienced an episode of SAM during follow-up were lighter and shorter at baseline than those who did not. The second hypothesis can be backed by prior literature suggesting differences in growth trajectories based on prenatal and early infancy influences (41, 42).

The hypotheses discussed above suggest that it might be unrealistic to expect that post-SAM children will ever fully catch up with the growth trajectory of non-wasted controls, especially when the deficit in length/height is large(43). However, it is worth mentioning that 14.4 % of the Post-SAM group experienced a complete reversal of stunting without any specific intervention. This percentage could have been higher if these children had received an intervention to promote linear catch-up growth, leading to ponderal catch-up. However, evidence on interventions that can promote linear catch-up growth after malnutrition or growth restriction is limited. Indeed, research on the topic has so far yielded contrasting results making it difficult to propose a specific or a package of interventions for this purpose (43-52). Thus, more research is needed to identify the best interventions for promoting growth catch-up following treatment of SAM.

Change in HAD is proposed as a better marker of linear catch-up than HAZ change (23, 53). In our study, HAZ and HAD trends led to the same conclusion in the total sample and in those who had stunting reversal suggesting that HAZ can be used to assess catch-up growth in the study setting. This result was observed despite inclusion of children above 24 months of age at baseline or those who crossed this age cut-off during the follow-up period. Thus, more studies are needed to demonstrate the benefit of HAD over HAZ in the evaluation of interventions' effectiveness on linear growth(53).

Our study had several strengths. First, the inclusion of comparison group that faced similar environmental and community contexts has allowed unbiased conclusions regarding the excess vulnerability of post-SAM children. The second strength was the prospective nature of the study that enhanced the accuracy of the collected data. Third, the monthly follow-up has minimised missing short episodes of AM. The lack of data on pre-SAM and treatment period growth parameters is the major limitation of the study. Availability of these data could have allowed a better understanding of the post-discharge growth pattern. Both admission and discharge criteria of the Ethiopian government at the time were different from what most CMAM programmes currently use, and this could limit generalizability. Finally, we don’t have biomarker data. As we proposed earlier, measurements including immune system reconstitution, correction of anaemia, and other micronutrient deficiencies would provide more reliable evidence of physiological and functional recovery.

In conclusion, this study has shown that post-SAM children remain at excess risk of AM and common disease than other children living in the same environment up to twelve months after graduating from treatment and that reoccurrence of SAM during this period should be considered differently than an episode diagnosed in a child without a history of SAM. Our results advocate for the design of post-discharge interventions that aim to prevent the reoccurrence of AM, reduce morbidity and promote catch-up growth. Further research is needed to improve the understanding of SAM episodes and SAM's effect on post-discharge growth patterns and to define the appropriate package of post-discharge interventions.

# Acknowledgements

We thank the mothers and children who consented to give their time and support freely throughout this study. We are grateful for the hard work of the field data collectors, supervisors and the data entry clerks for their essential contribution. We thank the ENGINE, Jimma University and Valid International management teams for their vast contribution to the supervision: Beyene Wondafrash, Habtamu Fekadu, Erika Lutz, Cherinet Abuye, Basia Benda and Anne Walsh.

# Funding

This was a partnership study for the USAID ENGINE programme in Ethiopia, implemented by Valid International, Jimma University and Save the Children. The research work was supported by United States Agency for International Development (USAID) under Agreement No. AID-663-A-11-00017. The contents of this paper are the sole responsibility of Valid International and Jimma University and do not necessarily reflect the views the funders. The funders had no role in study design, data collection and analysis, decision to publish, or preparation of the manuscript.

# Authors’ Contribution

| **Contributor Role** | Names |
| --- | --- |
| **Conceptualization** | H. Luo, K. Sadler, P. James, P. Bahwere, Tsinuel Girma |
| **Data Curation** | H. Luo, P. James, P Bahwere, Tsinuel Girma, Yesufe Getu, Yilkal Fantyae |
| **Formal Analysis** | Alemseged Abdissa, P. Bahwere, Tsinuel Girma, Yesufe Getu |
| **Funding Acquisition** | K. Sadler |
| **Investigation** | H. Luo, Alemseged Abdissa, P. James, Tsinuel Girma, Yesufe Getu |
| **Methodology** | H. Luo, Alemseged Abdissa, P. James, P. Bahwere, Tsinuel Girma |
| **Project Administration** | H. Luo, K. Sadler, Alemseged Abdissa, P. James, Tsinuel Girma, Yesufe Getu, Yilkal Fantyae |
| **Supervision** | H. Luo, K. Sadler, Alemseged Abdissa, P. Bahwere, Tsinuel Girma, Yesufe Getu, Yilkal Fantyae |
| **Writing – Original Draft Preparation** | P. Bahwere, Tsinuel Girma |
| **Writing – Review & Editing** | H. Luo, K. Sadler, Alemseged Abdissa P James, P. Bahwere, Tsinuel Girma, Yilkal Fantyae, Yesufe Getu |

# References.

1.	Sadler K, Myatt M, Feleke T, Collins S. A comparison of the programme coverage of two therapeutic feeding interventions implemented in neighbouring districts of Malawi. Public Health Nutr. 2007;10(9):907-13.

2.	Collins S, Sadler K, Dent N, Khara T, Guerrero S, Myatt M, et al. Key issues in the success of community-based management of severe malnutrition. Food Nutr Bull. 2006;27(3):S49-S82.

3.	Collins S, Dent N, Binns P, Bahwere P, Sadler K, Hallam A. Management of severe acute malnutrition in children. Lancet. 2006;368(9551):1992-2000.

4.	Rogers E, Myatt M, Woodhead S, Guerrero S, Alvarez JL. Coverage of community-based management of severe acute malnutrition programmes in twenty-one countries, 2012-2013. PLoS One. 2015;10(6):e0128666.

5.	Ciliberto MA, Sandige H, Ndekha MJ, Ashorn P, Briend A, Ciliberto HM, et al. Comparison of home-based therapy with ready-to-use therapeutic food with standard therapy in the treatment of malnourished Malawian children: a controlled, clinical effectiveness trial. Am J Clin Nutr. 2005;81(4):864-70.

6.	Banda T, Chawanda K, Tsuchida W, Kathumba S. Report of a Pilot Program Using a Milk-Free Ready-to-Use Therapeutic Food Made From Soya, Maize, and Sorghum to Treat Severe Acute Malnutrition. Food and Nutrition Bulletin. 2021;42(1):91-103.

7.	Bahwere P, Banda T, Sadler K, Nyirenda G, Owino V, Shaba B, et al. Effectiveness of milk whey protein-based ready-to-use therapeutic food in treatment of severe acute malnutrition in Malawian under-5 children: a randomised, double-blind, controlled non-inferiority clinical trial. Matern Child Nutr. 2014.

8.	Mengesha MM, Deyessa N, Tegegne BS, Dessie Y. Treatment outcome and factors affecting time to recovery in children with severe acute malnutrition treated at outpatient therapeutic care program. Global health action. 2016;9:30704.

9.	Stobaugh HC, Mayberry A, McGrath M, Bahwere P, Zagre NM, Manary MJ, et al. Relapse after severe acute malnutrition: A systematic literature review and secondary data analysis. Matern Child Nutr. 2018:e12702.

10.	WHO. Guideline: Updates on the management of severe acute malnutrition in infants and children. Geneva: World Health Organisation; 2013.

11.	Rytter MJ, Kolte L, Briend A, Friis H, Christensen VB. The immune system in children with malnutrition--a systematic review. PLoS One. 2014;9(8):e105017.

12.	Chevalier P, Sevilla R, Sejas E, Zalles L, Belmonte G, Parent G. Immune recovery of malnourished children takes longer than nutritional recovery: implications for treatment and discharge. J Trop Pediatr. 1998;44(5):304-7.

13.	Bahwere P, James P, Abdissa A, Getu Y, Getnet Y, Sadler K, et al. Use of tuberculin skin test for assessment of immune recovery among previously malnourished children in Ethiopia. BMC Res Notes. 2017;10(1):570.

14.	Lelijveld N, Seal A, Wells JC, Kirkby J, Opondo C, Chimwezi E, et al. Chronic disease outcomes after severe acute malnutrition in Malawian children (ChroSAM): a cohort study. The Lancet Global health. 2016.

15.	O'Sullivan NP, Lelijveld N, Rutishauser-Perera A, Kerac M, James P. Follow-up between 6 and 24 months after discharge from treatment for severe acute malnutrition in children aged 6-59 months: A systematic review. PLoS One. 2018;13(8):e0202053.

16.	Adegoke O, Arif S, Bahwere P, Harb J, Hug J, Jasper P, et al. Incidence of severe acute malnutrition after treatment: A prospective matched cohort study in Sokoto, Nigeria. Matern Child Nutr. 2020:e13070.

17.	Burza S, Mahajan R, Marino E, Sunyoto T, Shandilya C, Tabrez M, et al. Seasonal effect and long-term nutritional status following exit from a Community-Based Management of Severe Acute Malnutrition program in Bihar, India. Eur J Clin Nutr. 2016;70(4):437-44.

18.	Health Survey Central Statistical Agency, ICF International. Ethiopian Demographic and Health Survey 2011. Addis Ababa, Ethiopia and Calverton Maryland, USA2012.

19.	World Health Organization. Training Course on Child Growth Assessment. Geneva: WHO, 2008.[WHO website]. 2008.

20.	Ethiopia Federal Ministry of Health. Protocol for the management of severe acute malnutrition2007 2007.

21.	WHO. WHO child growth standards. Acta Paediatr. 2006;95(S450):1-101.

22.	Leroy J. ZSCORE06: Stata module to calculate anthropometric z-scores using the 2006 WHO child growth standards. Statistical Software Components. 2011.

23.	Leroy JL, Ruel M, Habicht JP, Frongillo EA. Using height-for-age differences (HAD) instead of height-for-age z-scores (HAZ) for the meaningful measurement of population-level catch-up in linear growth in children less than 5 years of age. BMC Pediatrics. 2015;15:145.

24.	Monteiro PO, Victora CG. Rapid growth in infancy and childhood and obesity in later life--a systematic review. Obes Rev. 2005;6(2):143-54.

25.	Cameron N. The human growth curve, canalization and catch-up growth. Human growth and development: Elsevier; 2012. p. 1-22.

26.	Pradeilles R, Norris T, Ferguson E, Gazdar H, Mazhar S, Bux Mallah H, et al. Factors associated with catch-up growth in early infancy in rural Pakistan: A longitudinal analysis of the women's work and nutrition study. Matern Child Nutr. 2019;15(2):e12733.

27.	Gonzales GB, Ngari MM, Njunge JM, Thitiri J, Mwalekwa L, Mturi N, et al. Phenotype is sustained during hospital readmissions following treatment for complicated severe malnutrition among Kenyan children: A retrospective cohort study. Matern Child Nutr. 2020;16(2):e12913.

28.	Akomo P, Bahwere P, Murakami H, Banda C, Maganga E, Kathumba S, et al. Soya, maize and sorghum ready-to-use therapeutic foods are more effective in correcting anaemia and iron deficiency than the standard ready-to-use therapeutic food: randomized controlled trial. BMC Public Health. 2019;19(1):806.

29.	Kangas ST, Salpeteur C, Nikiema V, Talley L, Briend A, Ritz C, et al. Vitamin A and iron status of children before and after treatment of uncomplicated severe acute malnutrition. Clin Nutr. 2020;39(11):3512-9.

30.	Sevilla R, Sejas E, Zalles L, Belmonte G, Chevalier P, Parent G, et al. ["CLAPSEN", a global approach to the rehabilitation of severe childhood malnutrition in Bolivia]. Sante. 2000;10(2):97-102.

31.	Chevalier P, Sevilla R, Zalles L, Sejas E, Belmonte G, Parent G, et al. [Immuno-nutritional recovery of children with severe malnutrition]. Sante. 1996;6(4):201-8.

32.	Berkley JA, Ngari M, Thitiri J, Mwalekwa L, Timbwa M, Hamid F, et al. Daily co-trimoxazole prophylaxis to prevent mortality in children with complicated severe acute malnutrition: a multicentre, double-blind, randomised placebo-controlled trial. The Lancet Global health. 2016;4(7):e464-73.

33.	Stobaugh HC, Bollinger LB, Adams SE, Crocker AH, Grise JB, Kennedy JA, et al. Effect of a package of health and nutrition services on sustained recovery in children after moderate acute malnutrition and factors related to sustaining recovery: a cluster-randomized trial. Am J Clin Nutr. 2017;106(2):657-66.

34.	Keats EC, Das JK, Salam RA, Lassi ZS, Imdad A, Black RE, et al. Effective interventions to address maternal and child malnutrition: an update of the evidence. Lancet Child Adolesc Health. 2021.

35.	Abdullahi AT, Farouk ZL, Imam A. Common mental disorders in mothers of children attending out-patient malnutrition clinics in rural North-western Nigeria: a cross-sectional study. BMC Public Health. 2021;21(1):185.

36.	Kerac M, Bunn J, Seal A, Thindwa M, Tomkins A, Sadler K, et al. Probiotics and prebiotics for severe acute malnutrition (PRONUT study): a double-blind efficacy randomised controlled trial in Malawi. Lancet. 2009;374(9684):136-44.

37.	WHO, WFP, ., UNICEF & UNSCN. Joint statement on the community-based management of severe malnutrition in children. 2007.

38.	Collins S. Treating severe acute malnutrition seriously. Arch Dis Child. 2007;92(5):453-61.

39.	Wells JC, Chomtho S, Fewtrell MS. Programming of body composition by early growth and nutrition. Proc Nutr Soc. 2007;66(3):423-34.

40.	Guilloteau P, Zabielski R, Hammon HM, Metges CC. Adverse effects of nutritional programming during prenatal and early postnatal life, some aspects of regulation and potential prevention and treatments. J Physiol Pharmacol. 2009;60 Suppl 3:17-35.

41.	Hediger ML, Overpeck MD, Maurer KR, Kuczmarski RJ, McGlynn A, Davis WW. Growth of infants and young children born small or large for gestational age: findings from the Third National Health and Nutrition Examination Survey. Arch Pediatr Adolesc Med. 1998;152(12):1225-31.

42.	Budree S, Stein DJ, Brittain K, Goddard E, Koen N, Barnett W, et al. Maternal and infant factors had a significant impact on birthweight and longitudinal growth in a South African birth cohort. Acta Paediatr. 2017;106(11):1793-801.

43.	Wang HH, Wei JR, Zhou WJ, Xu Q, Nie LH, Li L. [Nutritional recovery after discharge in hospitalized children with malnutrition]. Zhongguo Dang Dai Er Ke Za Zhi. 2020;22(8):882-6.

44.	Jee YH, Baron J, Phillip M, Bhutta ZA. Malnutrition and catch-up growth during childhood and puberty. World Rev Nutr Diet. 2014;109:89-100.

45.	Null C, Stewart CP, Pickering AJ, Dentz HN, Arnold BF, Arnold CD, et al. Effects of water quality, sanitation, handwashing, and nutritional interventions on diarrhoea and child growth in rural Kenya: a cluster-randomised controlled trial. The Lancet Global health. 2018;6(3):e316-e29.

46.	Luby SP, Rahman M, Arnold BF, Unicomb L, Ashraf S, Winch PJ, et al. Effects of water quality, sanitation, handwashing, and nutritional interventions on diarrhoea and child growth in rural Bangladesh: a cluster randomised controlled trial. The Lancet Global health. 2018;6(3):e302-e15.

47.	Bisimwa G, Owino VO, Bahwere P, Dramaix M, Donnen P, Dibari F, et al. Randomized controlled trial of the effectiveness of a soybean-maize-sorghum-based ready-to-use complementary food paste on infant growth in South Kivu, Democratic Republic of Congo. Am J Clin Nutr. 2012;95(5):1157-64.

48.	Das JK, Hoodbhoy Z, Salam RA, Bhutta AZ, Valenzuela-Rubio NG, Weise Prinzo Z, et al. Lipid-based nutrient supplements for maternal, birth, and infant developmental outcomes. Cochrane Database Syst Rev. 2018;8:CD012610.

49.	Goto E. Effectiveness of Prenatal Lipid-Based Nutrient Supplementation to Improve Birth Outcomes: A Meta-analysis. Am J Trop Med Hyg. 2019;101(5):994-9.

50.	Penny ME. Micronutrients in the treatment of stunting and moderate malnutrition. Nestle Nutr Inst Workshop Ser. 2012;70:11-21.

51.	Dearden KA, Schott W, Crookston BT, Humphries DL, Penny ME, Behrman JR, et al. Children with access to improved sanitation but not improved water are at lower risk of stunting compared to children without access: a cohort study in Ethiopia, India, Peru, and Vietnam. BMC Public Health. 2017;17(1):110.

52.	Ngari MM, Iversen PO, Thitiri J, Mwalekwa L, Timbwa M, Fegan GW, et al. Linear growth following complicated severe malnutrition: 1-year follow-up cohort of Kenyan children. Arch Dis Child. 2019;104(3):229-35.

53.	Frongillo EA, Leroy JL, Lapping K. Appropriate Use of Linear Growth Measures to Assess Impact of Interventions on Child Development and Catch-Up Growth. Adv Nutr. 2019;10(3):372-9.
