# Supplementary material for: Nutrition status and morbidity of Ethiopian children after recovery from severe acute malnutrition: Prospective matched cohort study
Source: PLoS One. 2022 Mar 10;17(3):e0264719. doi: 10.1371/journal.pone.0264719 (PMC8912152; doi:10.1371/journal.pone.0264719)
Supplement: Attachment — Submitted filename: Response to Reviewers_ Nov 15.docx [file pone.0264719.s006.docx]

**Point-by-point response**

The authors thank the Editor and both reviewers for taking their time to review our manuscript and provide us with valuable comments and suggestions.

A. Editor Comments
1. Please ensure that your manuscript meets PLOS ONE's style requirements, including those for file naming.

**The manuscript is checked for fitness to journal style.**

2. We note that you have indicated that data from this study are available upon request. PLOS only allows data to be available upon request if there are legal or ethical restrictions on sharing data publicly.

In your revised cover letter, please address the following prompts:

**The minimal anonymized data as Supporting Information file is now uploaded.**

3. We note that you have included the phrase “data not shown” in your manuscript. Unfortunately, this does not meet our data sharing requirements. PLOS does not permit references to inaccessible data. We require that authors provide all relevant data within the paper, Supporting Information files, or in an acceptable, public repository. Please add a citation to support this phrase or upload the data that corresponds with these findings to a stable repository (such as Figshare or Dryad) and provide and URLs, DOIs, or accession numbers that may be used to access these data. Or, if the data are not a core part of the research being presented in your study, we ask that you remove the phrase that refers to these data.

**We have now removed the specified phrase and uploaded the data as a Supporting Information file.**

4. 4. Please include captions for your Supporting Information files at the end of your manuscript, and update any in-text citations to match accordingly.

**Captions for our Supporting Information files are now included at the end of the manuscript and in-text citations are matched accordingly.**

5. Please review your reference list to ensure that it is complete and correct. If you have cited papers that have been retracted, please include the rationale for doing so in the manuscript text, or remove these references and replace them with relevant current references. Any changes to the reference list should be mentioned in the rebuttal letter that accompanies your revised manuscript. If you need to cite a retracted article, indicate the article’s retracted status in the References list and also include a citation and full reference for the retraction notice.

**The reference list is reviewed.**

5. Abstract: Please provide key statistical figures with confidence interval or p-values including the reported 15 times higher risk of developing SAM.

**The abstract is revised accordingly**.

6. Background
   Please expand the last paragraph on extent of SAM relapse rate after discharge. How common is the problem?

**Comment accepted and the manuscript is now revised accordingly.**

Methods

- Page 4, sample size estimation: “We projected an 8.5 difference…” per what population?

**This sentence is elaborated on in the revised version.**

- How did you ascertain that the controls had no episode of acute malnutrition?

**History of previous episode of acute malnutrition was excluded based on verbal confirmation from the primary caregiver.**

- How were the controls selected? I assume multiple age and sex matched controls would be available in the study villages.

**The mother/care taker of the case was asked to indicate the neighboring household having a child of the same sex and age. Study investigators followed up, and the child meeting the criteria closest to the case household was picked. We have now added these details to the manuscript.**

- The matching criteria for age is vague. I assume matching based on age was made based on some tolerable rage. What age range did you tolerate?

**The predefined age ranges were ±3 months up to 24 months of age and ±6 months for the older children. Only very few were out of the ±3 months range. We have now added these details to the manuscript.**

- It is not clear how growth trajectory was defined or calculated.

**We have defined under the study outcomes as ‘weight length/height, MUAC and related indices changes and trends’.**

7. Results

- Please add a table that compares the basic socio-demographic and nutritional characteristics of lost-to-follow (including deaths and early exits) and retained subjects for both groups so that it can help readers to understand the likelihood of loss to follow up bias.

**We have replaced the original table 1 with a new table 1 that includes comparison of the lost-to-follow up cases and retained cases by the study groups.**

- Table 1: I was expecting comparison based on basic socio-demographic variables including household wealth index or income and maternal educational status. Please include these variables in the list.

**Given that including all the parameters makes the main table too long, these additional data are given in Supplementary table 1.**

- Figure 2: some of the contents in the boxes are not visible
  **Figure 2 is now corrected.**

8. Discussion

- “Both admission and discharge criteria of the Ethiopian government at the time were different from what most CMAM programmes currently use, and this could limit generalizability”. This is an important point that requires clarification. What specific changes have been introduced to the protocol? How would that affect the generalizability of the study

**At the time of data collection: admission was based on MUAC <11cm.**

**Now: admission MUAC <11.5cm, WHZ <-3 SD or bilateral oedema and discharge when MUAC >= 11.5cm or WHZ >=-2 and absence of oedema.**

**The new admission criteria will increase the number of SAM children into treatment programmes, more children will start treatment in a relatively better physical state in terms of anthropometry, and they will stay longer on treatment getting RUTF. However, the impact of these changes may not be sufficient to prevent relapse or bring or maintain optimal growth and health during the first few months after discharge. Thus, we do not expect a major problem on the generalizability of our results.**

9. Conclusion

- What specific post-discharge interventions are you recommending for? Is that really financially and practically feasible to implement such longer supports to SAM cases? Is there any international experience before?

**The main point we want to make it that we need research into what the post-discharge package of care would look like. We weren’t necessarily advocating any specific intervention. We acknowledge the concerns regarding cost and feasibility of any future intervention that may be developed. At the same time, we recognize the need to add alongside this research - we should advocate for the economic and health costs of relapse. Then proper cost-effectiveness studies can be designed that compare different packages of post-discharge interventions with the savings made by averting relapse. We also suggested proper integration into existing growth monitoring programmes, which would not be unfeasibly expensive to maintain.**

B. Review Comments

Reviewer #1

10. How exactly were the controls carried out? It just says the selection criteria but how it was done in practice on the field? It would be better to say it here.

**Thank you. This comment has been addressed above and extra details added to the manuscript.**

11. It will be better to use the term “not exposed” instead of control

**In the study communities’ context, all the children are exposed to risk of undernutrition. We thus think that the term control is more appropriate than not-exposed.**

12. I think another limitation of this study is the fact that this study is unable to separate the effects of SAM from the effects of the physical, social and other environments in which these subjects evolve, especially since there are differences in the socio-economic and even dietary status of the two groups, as well as the status of their mothers (MUAC), as can be seen in Table 1 of the supplementary file

**By selecting children of the same communities/villages we did our best to ensure that these children would face the same environmental/climatic events, seasonal changes related to food production and local epidemiological/morbidity profile. We believe that we selected the best control group that we could in the context of a limited historical data on households and children characteristics.**

Reviewer #2

13. Please clarify if the SAM cases were uncomplicated, give some more information on their duration of treatment, and if the data are available, their starting MUAC or WHZ. The reason why this may be important is that children who began just below the threshold for SAM may more easily recover and have less relapse than children who began far below the threshold for SAM.

**We recruited the SAM cases immediately after their exit from the local CMAM programme, discharged as recovered cases as defined by the programme. Unfortunately, we did not have data of the cases during their admission and follow-up during treatment. However, we can safely assume that most if not all the cases were admitted into the CMAM as uncomplicated SAM, according to the national treatment protocol, since any children originally admitted to inpatient care would have been transferred to the outpatient CMAM programme when the cases were uncomplicated. We did not include any children coming direct from inpatient care.**

14. Under Methods, for ‘We projected an 8.5 difference between post-SAM and controls in incidence of acute malnutrition’ – what does the 8.5 refer to? Is it an absolute percentage difference, relative percentage, ratio? If a percentage difference, please more clearly state the estimated percentage in the control group with 95% CI.

**This comment has been addressed above.**

15. I am concerned by starting with an anticipated loss to follow up of 20% because this does not occur randomly. Children who become lost to follow up are usually more frequently ill, malnourished or have died. Please explain the rationale for 20%, efforts made to trace families and approach to dealing with the potential bias created. Fortunately, the actual figure was lower, but these issues remain.

**The 20% was set based on a literature review of the observed dropout rate in prospective studies in similar settings. Also, we wanted to maximize the study power. In the study area, many other factors than those mentioned by the reviewer can affect the rate of loss to follow up including relocation of the family to a village out if the study catchment area and isolation of the village during raining season. We believe that 20% was a conservative estimate of potential loss-to-follow-up, and thankfully this was sufficient for our study’s purpose.**

16. Please give more details of how controls were identified and recruited. They are usually more difficult to correctly enroll than cases. Location is not mentioned in matching – this may be fine but needs an explanation of how location was dealt with when enrolling controls. For example, if within the same woreda then this may be considered frequency matching.

**This has been addressed above. The controls were all found in the same village (a smaller entity than a woreda, the woreda corresponding to a district in other countries)**

17. In results, I suggest rewording ‘As shown in Table 1, no difference was observed between post-SAM and control groups for matching criteria at baseline except that the post-SAM children were significantly lighter and shorter (p<0.001)’ because weight and height were not matching criteria (only age and sex were mentioned in methods).

**Thank you, comment accepted. Revised accordingly.**

18. Likewise, ‘The burden of common morbidities was higher (p<0.001) among post-SAM than controls (Fig 3)’ should be more precisely worded than just the word ‘burden’ indicating in the text whether this refers to a rate, the number of events, or the proportion of children experiencing morbidity events.

**Comment accepted. Revised accordingly.**

19. Out of interest, were the post-SAM children who experienced accelerated linear growth younger? Given that the data were collected over time, it would be ideal to give the data on mortality and new episodes of malnutrition as rates, e.g. per 1000 child-years or 100 child-months.

**We agree this is interesting and had included this in table 4. The incidence rate among the post-SAM was 0.26 per 100 child-month of observation**

20. In the discussion, I would argue that correction of body mass deficit is not the primary objective of SAM treatment. The objectives are to reduce risks of mortality and illness, and to promote neurodevelopment. Improving body mass is one component of helping achieve that objective, but not the only one. Others include improving body composition, correcting micronutrient deficiencies, identifying and treating medical conditions, identifying and addressing contributory home circumstances, including maternal mental health. Focus on body mass gain may be among the reasons we continue to see mortality, relapse, failure of height growth etc.
    Might the apparent 14.4% reversal in stunting be due to regression to the mean or observation bias from loss to follow up?

**Though all the outcomes mentioned by the reviewer should ideally be achieved (and we agree with), correction of body mass deficit and survival are the primary treatment objectives of current treatment guidelines with CMAM and used as main programme indicators. Hence these remained our focus of the paper. However, in our original narrative we had written in the discussion that ‘other parameters should be considered for the decision on the optimal period of post-discharge follow-up, including immune system reconstitution, correction of anaemia, and other micronutrient deficiencies’. We also mentioned that ‘ assuming current research efforts are sustained, including testing new interventions such as those to address maternal mental disorders or environmental enteric dysfunction, there are chances that a package of post-treatment support to prevent the recurrence of acute malnutrition will be available shortly’. Thus we hope this text captures the reviewer’s nuances sufficiently.**

**Regarding the reversal of the stunting, we are not taking a normalization reference value but considered just crossing the cut-off. Thus, regression to the mean is not a concern in this case.**
